# Supplementary material for: Analysis of Answers to Queries among Anonymous Users with Gastroenterological Problems on an Internet Forum
Source: Int J Environ Res Public Health. 2020 Feb 6;17(3):1042. doi: 10.3390/ijerph17031042 (PMC7037061; doi:10.3390/ijerph17031042)
Supplement: Supplementary file 1 [file ijerph-17-01042-s001.pdf]

Supplementary File

Table S1

Working definitions of the most commonly reported symptoms by forum users.

No.	Symptoms	Definition
1.	Abdominal pain	Literally typed name or a synonym of the symptoms, or description of pain between the chest and pelvic region but no solely involving back.
2.	Diarrhoea	Literally typed name or a synonym of the symptoms, or description of loose or liquid bowel movements per day or at least three times defecation per day
3.	Bloating	Literally typed name or a synonym of the symptoms, or description of feeling a full, tight or distended abdomen.
4.	Nausea	Literally typed name or a synonym of the symptoms, or description of the unpleasant feeling of an urge to vomit.
5.	Constipation	Literally typed name or a synonym of the symptoms, or description of less than one stool per week or feeling of difficulty defecation requiring manual maneuvers to empty the rectum.
6.	Flatulence	Literally typed name or a synonym of the symptoms, or description of unpleasant and frequent gas passage through the anus.
7.	Intestinal rumbling	Literally typed name or a synonym of the symptoms, or description of embarrassing sounds from abdomen such as growling, gurgling, rumbling.
8.	Fatigue	Literally typed name or a synonym of the symptoms, or description of mental or physical exertion.
9.	Blood in stool	Literally typed name or a synonym of the symptoms, or description of reddened toilet paper or underwear or presence of tarry stool.
10.	Vomit	Literally typed name or a synonym of the symptoms, or description of involuntary, forceful expulsion of the stomach content through the mouth or the nose.
11.	Heartburn	Literally typed name or a synonym of the symptoms, or description of burning sensation in the chest or acid regurgitation to the oral cavity.
12.	Belching	Literally typed name or a synonym of the symptoms, or description of recurrent emitting gas from the gastrointestinal tract through the mouth.
13.	Rectal or anal pain	Literally typed name or a synonym of the symptoms, or description of pain or recurrent discomfort of the lower portion of the gastrointestinal tract which intensifies during anal receptive intercourse or defecation.
14.	Weight loss	Literally typed name or a synonym of the symptoms, or description of unintended weight loss more than 5% of weight during the last three months or more than 10% during the last six months.
15.	Loss of appetite	Literally typed name or a synonym of the symptoms, or description of the loss of desire to eat the meals that previously were tasty for the user.

Table S2

Univariate regression analysis. Dependent variable: obtaining at least one answer related to original poster problem (counted as one).

Independent variable	OR (95% CI)	p-value
Question about treatment of declared ailment	1.14 (0.97-1.33)	0.10
**Question about diagnostics of declared ailment**	**1.32 (1.12-1.55)**	**< 0.001**
**Request for diagnostics results interpretation**	**0.47 (0.36-0.61)**	**< 0.001**
Request for drug/doctor/healthcare facility recommendation [diagnosed disease]	0.75 (0.49-1.14)	0.18
Request for emotional support and/or motivation	1.32 (0.92-1.87)	0.13
**Complexity of the problem**	**1.32 (1.13-1.53)**	**< 0.001**
**Duration of the problem (> four weeks)**	**1.26 (1.07-1.49)**	**< 0.01**
**Abdominal pain**	**1.16 (0.99-1.36)**	**0.058**
Ascites	1.13 (0.38-3.37)	0.83
Anus itch	1.62 (0.87-3.04)	0.13
Anus lesion	0.97 (0.48-1.94)	0.93
Back pain	0.76 (0.46-1.25)	0.28
Belching	1.01 (0.71-1.43)	0.96
**Bloating**	**1.30 (1.04-1.63)**	**0.02**
**Blood in stool**	**1.85 (1.37-2.51)**	**< 0.001**
Chest pain	0.82 (0.47-1.44)	0.49
**Constipation**	**1.62 (1.27-2.07)**	**< 0.001**
**Diarrhea**	**1.49 (1.21-1.82)**	**< 0.001**
Dyspepsia	1.09 (0.70-1.69)	0.72
Dysphagia	1.05 (0.42-2.68)	0.91
Fatigue	1.19 (0.88-1.60)	0.25
**Feeling of incomplete defecation**	**2.61 (1.47-4.63)**	**< 0.001**
Fever	1.11 (0.57-2.17)	0.76
**Flatulence**	**1.45 (1.12-1.88)**	**< 0.01**
Halitosis	1.37 (0.82-2.30)	0.23
**Headache**	**1.66 (1.01-2.72)**	**0.045**
Heartburn	1.03 (0.73-1.44)	0.88
Hemoptysis	3.31 (0.64-17.07)	0.13
Hypogastrium region pain	1.31 (0.91-1.89)	0.14
Intestinal rumbling	1.22 (0.93-1.61)	0.16
Jaundice	1.32 (0.27-6.55)	0.74
Left lower quadrant pain	1.03 (0.66-1.61)	0.89
Light upper quadrant pain	1.08 (0.78-1.49)	0.64
**Loss of an appetite**	**1.43 (0.94-2.18)**	**0.098**
Nausea	0.94 (0.74-1.19)	0.59
Odynophagia	0.66 (0.16-2.64)	0.55

**Rectal or anal pain**	**1.84 (1.22-2.77)**	**< 0.01**
Right lower quadrant pain	0.92 (0.60-1.39)	0.68
Right upper quadrant pain	1.12 (0.83-1.51)	0.48
Subfebrile	0.81 (0.41-1.63)	0.56
Umbilical region pain	1.13 (0.78-1.62)	0.53
Vomit	1.03 (0.74-1.43)	0.87
Weight loss	1.12 (0.74-1.69)	0.60
Weight gain	0.66 (0.26-1.63)	0.30
Anal fissure	2.64 (0.48-14.45)	0.25
Anemia	0.38 (0.08-1.81)	0.18
Anxiety disorder	0.88 (0.31-2.47)	0.81
**Arterial hypertension**	**9.28 (1.14-75.51)**	**< 0.01**
**Bile reflux**	**0.16 (0.02-1.31)**	**0.040**
Biliary tract diseases	0.44 (0.12-1.62)	0.19
Celiac disease	1.10 (0.33-3.61)	0.88
Chronic pancreatitis	0.44 (0.05-4.23)	0.45
Depression	0.99 (0.22-4.43)	0.99
Diabetes	0.56 (0.15-2.19)	0.39
**Duodenitis**	**0.35 (0.12-1.06)**	**0.042**
Esophageal varices	0.44 (0.05-4.23)	0.45
Esophagitis	0.99 (0.22-4.43)	0.99
Gastroenteritis	0.78 (0.52-1.19)	0.25
Gastrointestinal allergies and intolerances	0.60 (0.21-1.72)	0.33
Gastrointestinal fungal disease	1.05 (0.28-3.94)	0.94
Gastroesophageal reflux disease	0.26 (0.03-2.25)	0.17
Helicobacter pylori infection	0.91 (0.50-1.67)	0.76
Hemorroids	1.27 (0.70-2.28)	0.43
Hiatal hernia	1.13 (0.60-2.13)	0.70
Hypothyroidism	0.71 (0.28-1.78)	0.46
Inflammatory bowel disease	0.66 (0.31-1.41)	0.27
**Irritable bowel syndrome**	**1.65 (1.01-2.68)**	**0.044**
Liver cirrhoris	0.56 (0.15-2.19)	0.39
Malignancy	0.66 (0.06-7.27)	0.73
Overweight or obese	1.76 (0.61-5.09)	0.29
Parasites	0.75 (0.22-2.58)	0.64
Pregnancy	0.61 (0.25-1.51)	0.28
Peptic ulcer disease	0.58 (0.25-1.35)	0.19
Viral hepatisis	0.33 (0.07-1.55)	0.12

CI – Confidence Interval; OR – Odds Ratio

Table S3

Examples of threads from the gastroenterology section of the Polish forum for anonymous users. The author's comments are presented under the conversations.

A. Record ID 254

Polish (original spelling)	English translation
Tytuł: Problemy żołądkowe	Title: Constipation with weight loss
Initial post (OP)<br><br>Od okolo 3 miesiecy mam problemy zoladkowe.<br>Mam wzdecia z wiatrami, mdlosci zaparcia, bóle w dołku podsercowym, a ostatnio pojawiły się silne bóle żeber po obu stronach. Towarzysza mi dziwne odgłosy w jelitach - bulgotanie i nieustające bóle i zawroty głowy. Zauwazylam takze spadek wagi ok.5 kg zauwazylam takze dwukrotnie krew w stolcu, byla to swieza krew o barwie jasno-czerwonej, ktora zauwazylam na papierze toaletowym... morfologie mam w porzadku, badania moczu takze, proby watrobowe i trzustkowe tez, badanie gastroskopowe tez nic nie wykazalo ,,prosze pomozcie !!!	Initial post (OP)<br><br>I have had stomach problems for about 3 months.<br>I have flatulence, nausea, constipation, chest pain and recently I have been feeling severe pain in the ribs on both sides. I have strange noises in my gut - gurgling and constant headaches and dizziness. I also noticed a weight loss of about 5 kg. I also found the blood in the stool twice. It was fresh blood with a light red color, which I noticed on toilet paper. I performed full blood count and it was alright, as well as urine tests, liver, and pancreatic tests. Gastroscopy didn't show any abnormalities too. Please help !!!
SubOP1<br><br>Witam Czy pojawiają się też zaburzenia w rytmie wypróżnień (biegunki, zaparcia)? Czy oprócz krwi w stolcu widoczny był śluz? Oraz przede wszystkim ile masz lat? Pozdrawiam	SubOP1<br><br>Hello. Do you have any problems with the frequency of bowel movements (diarrhea, constipation)? Was mucus visible in addition to blood in the stool? And above all, how old are you? Best regards
OP2 (OP response)<br><br>mam 20 lat, tak w stolcu pojawia sie sluz, na poczatku wystepowaly tylko biegunki, pozniej na przemian zaparcia z biegunkami, a teraz pojawiaja sie tylko zaparcia. zazwyczaj	OP2 (OP response)<br><br>I am 20 years old. Yes, mucus appears in the stool. In the beginning, there I had only diarrhea. Later, I had alternately constipation with diarrhea, and now only constipation appears. I had usually painful pressure on the stool ....

wystepuje bolesne parcie na stolec	
OP3 (OP update)<br><br>jezeli to ma znaczenie to moj stolec jest na<br>przemian barwy ciemnej (prawie czarnej) bądź jasnej jasno brązowa bądź zolta)	OP3 (OP update)<br><br>If that matters, my stool is alternately dark (almost black) and light brown or yellow)
SubOP2<br><br>Świeżo-czerwona krew może być związana zpodrażnieniem skóry odbytu papierem toaletowym lub obecnością hemoroidów, jednak dolegliwość ta w twoim wieku jest wyjątkową rzadkością. Objawy takie u młodej osoby mogą też pojawiać się w nieswoistych zapaleniach jelit, aby je zdiagnozować należałoby wykonać kolonoskopię, ale nie wiem, czy jest sens męczyć Cię tym badaniem - tutaj decyzja powinna należeć do lekarza, który zlecał pozostałe badania. Być może Twoje dolegliwości to objaw tzw czynnościowych zaburzeń przewodu pokarmowego - zespołu jelita wrażliwego, dyspepsji czynnościowej i mają związek ze stresem. Pozdrawiam	SubOP2<br><br>Fresh red blood may be associated with anal skin irritation with toilet paper or the presence of hemorrhoids. However,<br>this complaint is unique rare at your age. These symptoms can affect a young person<br>also in inflammatory bowel disease. Perhaps, you should perform a colonoscopy, but I don't know if it makes sense to<br>bother you with this test - the doctor who commissioned other tests should decide. Maybe your problems are a symptom of so-called functional gastrointestinal disorders such as sensitive bowel syndrome, functional dyspepsia and are associated with stress. Best regards
OP4 (OP update)<br><br>lekarz, ktory zlecal mi badania oddal mi wszystkie wyniki i kazal zglosic sie do poradni gastrologicznej ... a ja nie wiem juz co z tymi dolegliwosciami zrobic....	OP4 (OP update)<br><br>The doctor who ordered the tests gave me all the lab results and told me to go to the gastrological clinic. I don't know what to do with my ailments.
SubOP3<br><br>Jedynym wyjściem jest zgodnie z poleceniem lekarza zgłoszenie się do poradni gastrologicznej, aby móc poszukiwać przyczyny problemu. Do tego czasu warto zadbać o	SubOP3<br><br>As the doctor recommends, the only way out is to go to a gastroenterology clinic to search for the cause of the problem. Until then, it is worth ensuring a proper diet and regular consumption of meals at fixed

odpowiednią dietę i regularne spożywanie posiłków o stałych porach, jeśli dominują zaparcia - jeść dużo owoców i warzyw, być aktywnym fizycznie. Warto ograniczyć stres, dolegliwości ze strony przewodu pokarmowego często ulegają nasileniu pod wpływem napięcia emocjonalnego. Pozdrawiam	times. If constipation predominates - eat a lot of fruit and vegetables, be physically active. It is worth to reduce stress. Gastrointestinal problems often get worse under the influence of emotional tension. Best regards
SubOP4 (subOPstory)<br><br>witam mam 21 lat i od dłuższego czasu nie mogę pozbyć się wzdęć mój brzuch wygląda jak balon;/ w dodatku mam szczupłą budowę ciała przeszło 2 lata temu miałam problemy z wątrobą nie wie czy ma to teraz jakieś znaczenie leki typu espumisan itp nie pomagają w dodatku nie narzekam na brak ruchu i w miarę zdrowo się odżywiam...nie wiem co z tym zrobić prosze o odpowiedz	SubOP4 (subOPstory)<br><br>Hello, I'm 21 years old and for a long time, I can't get rid of bloating. My stomach looks like a balloon. Besides, I am slim and over 2 years ago I had problems with the liver. I don’t know if it has any importance now. Some drugs like espumisan etc. do not help. I don't complain about the lack of exercise and I eat well enough ... I don't know what to do with it, please answer.
SubOP5 (subOPstory)<br><br>ja te objawy co opisalam wyzej te co towarzysza mi od 3 miesiey.... udalam sie do gastroenterologa i lekarz przepisal i vermox na pasozyty i zalozyl diagnoze jelita drazliwego i przepisal 2 opakowania Duspatalinu... objawy nie przeszly.... wrecz jest gorze klucie w brzuchu i wiatry sie nasilily. towarzysz mi dosłownie co chwile ;/ bol nasila sie przy siadaniu. juz nie wytrzymuje nie wiem juz co mam robic niech mi pan cos doradzi....	SubOP5 (subOPstory)<br><br>I have these symptoms as described above for 3 months. I saw a gastroenterologist and the doctor prescribed vermox for parasites and diagnosed irritable bowel syndrome. The doctor prescribed 2 packages of Duspatalin too. The symptoms have not gone away. It is even worse. Stomach ache and gases intensified and it troubles me literally all the time. The pain intensifies when I sit down. I can't stand it anymore, I don't know what to do anymore. Give me some advice, please.

OP – original poster, subOP – suboriginal poster

The Authors comment:

In this case, OP presented some gastrointestinal problems such as flatulence, nausea, constipation, intestinal rumbling, alternately diarrhea and constipation, bloody stool with

mucus as well as the pain of the chest, head and in addition, dizziness with weight loss. Tests performed by OP did not show any abnormalities.

Responders asked the OP for more information about his symptoms. Firstly, subOPs recommended seeing a gastroenterologist, and secondly, changing diet or reducing stress. Furthermore, other forum members looked for help by writing about their problems which were similar to those that the OP's had, but they did not find an answer.

This example presents a typical post concerning gastrointestinal problems where the OP is just given the advice to go to see a doctor. This recommendation is rational because the differentiation of this condition should be done by a professional.

B. Record ID 2038

Polish (original spelling)	English translation
Tytuł: Problem z dwunastnicą	Title: Problem with duodenum
Initial post (OP)<br><br>Witam! Byłem na gastroskopii i postawiono diagnozę: ZNIEKSZTAŁCENIE I OWRZODZENIE OPUSZKI DWUNASTNICY. INFEKCJA HP. Pan który wykonywał badanie chyba był wyłącznie od tego bo nie udzielił mi żadnej informacji, jak leczyć, jakie są tego przyczyny itd. Wnioskuję, że muszę iść do specjalisty, ale chciałbym Was zapytać czy to poważna sprawa? Co można jeść, a czego unikać? Chciałbym jakichkolwiek informacji na ten temat! Z góry bardzo dziękuję! pozdrawiam!	Initial post (OP)<br><br>Welcome! I had a gastroscopy and the diagnosed was made: DEFORMITY AND ULCERATION OF DUODENAL BULB, HP INFECTION. The man who performed the examination didn't give me any information about the treatment and causes of this condition. I presume, that I should see a specialist but I want to ask you if I have a serious problem. What can I eat and what I shouldn't? I'd like to get to know anything about this subject. Thank you in advance! Best regards!
SubOP1<br><br>Musi Pan przejść kurację antybiotykową i dodatkowo stosować leki z grupy inhibitorów pompy protonowej. Leczenie tymi drugimi może być długotrwałe, ale objawy powinny szybko ustąpić. Na pewno czeka Pana powtórka gastroskopii (za 3-6 miesięcy). Lekarz wykonujący badanie nie udziela informacji o wynikach badania, bo to wchodzi w zakres porady (konsultacji), a nie badania diagnostycznego.	SubOP1<br><br>You must undergo antibiotic treatment and additionally use drugs from the group of proton pump inhibitors. Treatment with the proton pump inhibitors may be long-lasting, but the symptoms should disappear quickly. You will definitely have to repeat the gastroscopy (in 3-6 months). The doctor performing the test does not provide information on the results of the examination, because you should be informed about it during the consultation, not a diagnostic examination.

OP – original poster, subOP – suboriginal poster

In this example, the OP asked about the interpretation of the result of gastroscopy. The OP knew that he should have visited the specialist but he wanted to know if his problem is serious. Forum member explained that he has to start the treatment, as well as that he has to perform follow-up gastroscopy. The OP was also informed why the doctor performing the test, did not give any information about the results.

This tip seems to be somewhat rational. SubOP described the most important treatment – the eradication of H. pylori. However, repeated gastroscopy is unnecessary unlike in peptic ulcers in the stomach. Moreover, the subOP did not provide any information on lifestyle.

This short example presents using the forum to get diagnostic interpretation before consultation with the professional and the risk of misleading information. The OP may be confused by two different opinions on follow-up gastroscopy. We hope that the specialist authority restrains the user from the unnecessary procedure.

C. Record ID 248

Polish (original spelling)	English translation
Tytuł: Przewlekłe nieswoiste zmiany zapalne jelita	Title: Chronic non-specific intestine inflammation
Initial post (OP)<br><br>Witam serdecznie,Jestem stosunkowo młodą osobą - 24 lata. Problemy żołądkowe miewam często i szczerze mówiąc odkąd pamiętam, na przemian okresy biegunek i zatwardzeń. Miałam zrobioną kolonoskopię (przy wypróżnianiu często pojawiała się świeża krew) i okazało się, że mam przewlekłe nieswoiste zmiany zapalne jelita - chciałbym dowiedzieć się czy to coś poważnego i jak wygląda leczenie? Z góry bardzo dziękuje za odpowiedzi. Pozdrawiam Wiktoria	Initial post (OP)<br><br>Hello, I am a relatively young person - 24 years old. I have stomach problems often and honestly, since I can remember, alternating periods of diarrhea and constipation. I had a colonoscopy (fresh blood often appeared during defecation) and it turned out that I had chronic inflammatory bowel disease - I would like to know if this is serious and what does the treatment look like? Thank you in advance for your answers. Best regards, Victoria
SubOP1<br><br>jak na mój gust trzeba podziałać chemoprewencyjnie, rozmawiałaś z lekarzem o asamaxie? podobno bardzo dobry lek i wielu ludzi o nim pisze na forach, życzę zdrowia i nie łam się	SubOP1<br><br>I guess you need chemoprevention. Did you talk to your doctor about asamax? Apparently, this is very good medicine and many people talk about it on forums. I wish you were healthy, and don't break down
SubOP 2<br><br>Zapalenie wrzodziejące jelita grubego - bo o nim tutaj mowa - jest chorobą przewlekłą i jej przebieg jest sprawą bardzo indywidualną. Choroba może przebiegać bardzo łagodnie lub, co jest rzadkie w naszej szerokości geograficznej i u Polaków - bardzo burzliwie, do stanów zagrażających życiu włącznie. Zwykle pierwsze lata choroby są najgorsze, tak jak Pani opisuje - biegunka, krew w stolcu, później - najczęściej choroba się "wycisza" i przeplatana jest okresami	SubOP 2<br><br>Colitis ulcerosa - because we are talking about it here - is a chronic disease and its course is a very individual. The disease can be very mild or, which is rare in our latitude and in Poles - very stormy, including life-threatening conditions. Usually, the first years of illness are the worst, as you describe - diarrhea, blood in the stool, later - most often the disease "calms down" with alternated periods of malaise or recurrence of symptoms. You will have to use medicines, probably

gorszego samopoczucia lub nawrotu objawów. Leki będzie musiała Pani stosować przez czas nieokreślony, prawdopodobnie bez przerwy do końca życia. W okresach zaostrzeń - biegunek z krwią stosuje się mesalazynę ASAMAX 2 tabletki 3 razy na dobę, jeżeli to nie wystarcza - dodatkowo sterydy lub leki immunosupresyjne. W okresie remisji ASAMAX od 3 do 4 tabletek na dobę. Koszt tego leczenia obecnie nie jest taki duży (leki są refundowane, z odpłatnością ryczałtową). Kontrolne kolonoskopie raz na kilka lat (o ile nie wystąpią dodatkowe objawy), a po 10 latach trwania choroby - raz na rok, każdorazowo z pobraniem wycinków. Młody wiek w przypadku tej choroby potwierdza diagnozę. Podczas kolonoskopii powinni pobrać Pani wycinki do badań hist.-pat. (procedura standardowa).Jeżeli tego nie zrobili - popełnili błąd w sztuce.	without interruption for the rest of your life. During periods of exacerbation - diarrhea with blood, 2 tablets of mesalazine - ASAMAX 3 times a day should be used, if this is not enough - additionally steroids or immunosuppressants. During the remission period, 3 to 4 tablets of ASAMAX should be taken a day. The cost of this treatment is not so high at the moment (medicines are reimbursed with a flat-rate fee). Control colonoscopies every few years (unless additional symptoms occur), and after 10 years of illness - once a year, each time with biopsy. The young age for this disease confirms the diagnosis. During a colonoscopy, samples for the histopathological examination should be collected. (standard procedure). If they did not do it - it was medical malpractice.
OP2 (OP update)<br><br>Serdecznie dziękuję za odpowiedzi. Wycinki do badań pobrano i po odbiorze wyników była właśnie diagnoza - przewlekłe nieswoiste zmiany zapalne jelit. Z wynikami zjawiłam się u mojego chirurga, gdyż to on zlecił kolonoskopię (bo nie stwierdził hemoroidów, a krew była/nadal jest), teraz dostałam kolejne skierowanie tylko, że do proktologa czy słusznie?	OP2 (OP update)<br><br>Thank you very much for the answers. The biopsy was performed and the diagnosis was received - chronic inflammatory bowel disease. With the results, I came to my surgeon, because he ordered a colonoscopy (because he did not find hemorrhoids, and the blood was / still is in the anal area). Now, the other doctor (proctologist) ordered another colonoscopy. Is it right?
SubOP3<br><br>Nieswoistym zapaleniem jelit zajmuje się gastroenterolog i do niego powinna Pani zostać skierowana, nawet jeśli zmiany zapalne jelit są ograniczone tylko do odbytnicy - chociażby dlatego,	SubOP3<br><br>A gastroenterologist deals with inflammatory bowel disease, so you should be referred to a gastroenterologist, even if the inflammatory bowel lesions are limited

że w części przypadków choroba może się szerzyć na całe jelito grube, a proktolog właściwie zajmuje się tylko odbytnicą.	to the rectum - because in some cases the disease can spread to the entire large intestine, and the proctologist actually only deals with the rectum.
OP3 (OP update)<br><br>Takie skierowanie dał mi chirurg, powiedział, że proktolog jest adekwatny do tych spraw... Nie znam się niestety na tym wszystkim. Rozumiem, że jednak nieswoiste zmiany zapalne jelit muszą być leczone i nie wolno tego lekceważyć, bo tego też lekarz mi nie powiedział otwarcie... Czyli tak naprawdę wizyta u proktologa nic mi nie da?	OP3 (OP update)<br><br>I was given such a referral by a surgeon. He said, that the proctologist is adequate to these matters ... Unfortunately, I do not know all this. I understand that non-specific inflammatory bowel lesions must be treated and must not be underestimated, because the doctor didn't tell me about it openly ... So really, the proctological appointment will not give me anything?
OP 4 (OP update)<br><br>W każdym razie jeszcze raz BARDZO dziękuję za informację, jest mi to bardzo pomocne, bo jak do tej pory żaden lekarz się nie wysilił by mi cokolwiek wyjaśnić.	OP 4 (OP update)<br><br>Anyway, once again I want to say VERY thank you for the information, it is very helpful for me because so far no doctor has made an effort to explain anything to me.

OP – original poster, subOP – suboriginal poster

The main problem of the OP was alternate constipation and diarrhea with blood in the stool. Additionally, the colonoscopy showed chronic non-specific intestine inflammation. The OP was also referred to as a proctologist by the surgeon and considers if it was a good decision.

The responders explained that the result of colonoscopy pointed to the diagnosis of colitis ulcerosa and described the disease. They also proposed visiting a gastroenterologist instead of a proctologist and told about possibilities of the treatment with mesalazine, steroids or immunosuppressive drugs. These tips were very detailed and concerned even the dosage.

This case showed that the medical forum can clarify some issues about the serious disease. Nonetheless, information about drug dosage should be given only by a specialist and can cause confusion when it is communicated by a random, probably non-qualified person.

D. Record ID 2187

Polish (original spelling)	English translation
Tytuł: Burczenie w brzuchu przyczyny	Title: Intestinal rumbling - causes
Initial post (OP)<br><br>Często mi burczy w brzuchu nawet zaraz po jedzeniu. Czym to może być spowodowane? Mam nadzieję że to mi przejdzie, bo bardzo mnie to krępuje. Boję się jednak iść z tym do lekarza bo koleżanka straszyła, że będę miała robioną gastroskopię. Co mam robić? Proszę o pomoc.	Initial post (OP)<br><br>My stomach often rumbles even after eating. What can cause this? I hope that the problem will disappear because I feel very embarrassed. However, I'm afraid to go to the doctor because my friend threatened that I would have a gastroscopy. What should I do? Please help.
SubOP 1<br><br>gastroskopia nie jest niczym strasznym a wręcz bardzo potrzebnym i koniecznym w niektórych przypadkach, a czy miewasz przy okazji bóle brzucha? jaki masz kał? podajesz za mało szczegółów	SubOP 1<br><br>Gastroscopy is not terrible and it is even necessary in some cases. Anyway, do you have any stomach pains? How looks your feces? You don't provide enough details
SubOP 2<br><br>Gastroskopia? a co powiesz o kolonoskopii? ja biorę asamax od 3 lat i mam to badanie co roku	SubOP 2<br><br>Gastroscopy? What about a colonoscopy? I have been taking asamax for 3 years and I have this test every year
SubOP 3<br><br>asamax czyli mesalazyna?!	SubOP 3<br><br>asamax is it the same as mesalazine?!
SubOP 4<br><br>Burczenie w brzuchu związane jest z przemieszczaniem się znajdujących się w jelitach gazów. Zwiększona ilość gazów powstaje, gdy ktoś spożywa pewne pokarmy - znane jest działanie w tym względzie cebuli, fasoli czy grochu, ale nie każdy wie, że ilość gazów może ulegać zwiększeniu u osób spożywających dużą ilość batonów oraz innych słodyczy. Zwiększenie gazów może również wystąpić u osób, które dużo mówią	SubOP 4<br><br>The intestinal rumbling is associated with the movement of gases in the intestines. An increased volume of gas is created when someone eats certain foods - the action of onions, beans or peas is well known, but not everyone knows that the amount of gas can be increased in people consuming a large number of bars and other sweets. Increased gas volume can also occur in people who talk a lot while eating or eat quickly - in these cases, involuntary air swallowing

podczas jedzenia lub szybko jedzą - dochodzi w takich przypadkach do mimowolnego połykania powietrza. Rada na zmniejszenie burczenia jest następująca - jeść powoli, w miarę możliwości ograniczać rozmowę, unikać słodyczy, spożywać dużo warzyw i owoców. Spożywanie warzyw i owoców zwiększa ilość błonnika w przewodzie pokarmowym, a to z kolei daje lepsze wypełnienie treścią pokarmowa i chroni przed występowaniem gazów. Jeżeli oprócz burczenia w brzuchu miewasz wzdęcia, zaparcia lub biegunki - możliwe, że cierpisz na nadwrażliwe jelito. Wtedy konieczna jest zmiana diety, ponieważ z tym schorzeniem same leki sobie nie poradzą.	occurs. The advice on reducing grumbling is - eat slowly, limit talking as much as possible while eating, avoid sweets, eat lots of vegetables and fruits. Eating fruit and vegetables increases the amount of fiber in the digestive tract, which gives better gastrointestinal content and prevents gas formation. If you have bloating, constipation or diarrhea in addition to your stomach rumbling - you may have an irritable bowel syndrome. Then, it is necessary to change the diet, because, in this disease, drugs are not enough.
SubOP 5<br><br>Mnie też burczy w brzuchu. Stosuję dietę i nie jem cukru i produktów wzdymających i dalej lipa. Byłem z tym u gastrologa, dostałem xifaxan po którym miałem srakę. Pomaga mi jak się najem na noc czosnku. Więc chyba ta przypadłość powodowana jest przez jakieś bakterie. Czy ma ktoś jakiś pomysł?	SubOP 5<br><br>My stomach is rumbling too. I follow a diet and do not eat sugar and bloating products and nothing helps. I visited a gastroenterologist. He prescribed xifaxan, and after that, I had the trots. It helps me when I eat garlic for the night. So, probably this ailment is caused by some bacteria. Does anyone have any idea?
SubOP 6<br><br>Pomaga ci siemię lniane? Bo mi tak, ale tymczasowo. Ono chyba pomaga zregenerować błonę śluzową żołądka, jeszcze słyszałem coś o dobrym działaniu naparu z tataraku. Ale sam mam z tym kłopoty i nie wiem jak to całkowicie wyleczyć na dłuższy okres. Cały czas poszukuję rozwiązania	SubOP 6<br><br>Does linseed help you? Because it helps me, but only temporarily. It probably helps regenerate the gastric mucosa, I have heard something about the good effect of the calamus brew. But I'm in trouble and I don't know how to cure my ailment for a long time. I am still looking for a solution.
OP<br><br>Len - trochę pomaga. Ja nie mam biegunek czy zaparć. Nic mnie nie boli. Po prostu burczy mi w brzuchu. Spróbuję jeszcze cynamonu a jak nie to wracam do	OP<br><br>Linseed - it helps a little. I don't have diarrhea or constipation. Nothing hurts me. I have just intestinal rumbling. I will try out the cinnamon and if it doesn't help, I will

xifaxanu. Wydaj mi się, że przyczyną są bakterie albo jakieś świństwo / Gastrolog mówił mi że trzeba kilka razy powtórzyć kurację antybiotykową	take xifaxan again. It seems to me that the cause is bacteria or some other bloody thing / Gastroenterologist told me that I have to repeat the antibiotic treatment several times
SubOP 7<br><br>wszyscy mają prawie to samo, tyle ludzi to czyta, czy nikt nie wie jak temu zaradzić?	SubOP 7<br><br>Everybody has almost the same problem, so many people read it, does nobody know how to deal with it?
SubOP 8<br><br>3 razy dziennie łyżeczka cynamonu, żadnych surowych warzyw i smażonego. Narazie jest ok	SubOP 8<br><br>3 times a day I eat a teaspoon of cinnamon. I don't eat any vegetables and I don't fry anything. It's okay so far.
SubOP 9<br><br>na burczenie doostałem od lekarza duspatalin retard i po 2 tabletkach jest poprawa. Podobno ja ekest trzeba czekać dopiero 7 dni ale na razie	SubOP 9<br><br>for the intestinal rumbling, my doctor prescribed me duspatalin retard and after 2 tablets there is an improvement. People say that for the effect I have to wait 7 days, but for now
SubOP 10<br><br>Teraz do szkoły zamiast o 6.30 wstaję wcześniej (np. 5.30) i coś zjadam ;P później czuję się znacznie lepiej nawet nie burczy mi tak głośno w brzuchu, może troche, ale minimalnie, żuję również gumę miętową i przed wyjściem staram się czegoś napić : jogurt, woda itp)	SubOP 10<br><br>Now, when I wake up to school instead of at 6.30 I get up earlier (e.g. 5.30) and eat something ;P later, I feel much better, it doesn't even rumble so loudly in my stomach, maybe a little, but minimally. I also chew mint chewing gum and try to drink something before leaving: yogurt, water, etc.)
SubOP 11<br><br>"Miałem srakę" mnie rozwaliło.	SubOP 11<br><br>"I had the trots" knocked me down
SubOP 12<br><br>Ja gdy mam wzdęcia to w ostateczności biorę espumisan, tak to piję zieloną herbatę, duże ilości wody. Ważne jest też	SubOP 12<br><br>When I have flatulence, I take espumisan as a last resort. Usually, I drink green tea, large amounts of water. It is also important to be

aby umieć słuchać swój organizm. Ja gdy czuję że mogę mieć wzdęcia to nie jem fasoli, kalafiora i innych potraw wzdymających. Słyszałam też, że sałata pomaga w odgazowywaniu	able to listen to your body. When I feel that I may have flatulence, I don't eat beans, cauliflower, and other bloating dishes. I have also heard that lettuce helps to have less amount of gases
SubOP 13<br><br>W pozbywaniu się powietrza z układu pokarmowego pomaga również ruch typu brzuszki. Ponadto rzeczywiście jeśli ma się stateczny tryb życia warto mieć pod ręką espumisan. No i woda, dużo wody.	SubOP 13<br><br>Doing sit-ups also help to get rid of air from the digestive system. In addition, if you have a stable lifestyle, you should keep espumisan around. And water, a lot of water.
SubOP 14<br><br>Mnie też burczy w brzuchu. Stosuję dietę i nie jem cukru i produktów wzdymających i dalej lipa. Byłem z tym u gastrologa, dostałem xifaxan po którym miałem srakę. Pomaga mi jak się najem na noc czosnku. Więc chyba ta przypadłość powodowana jest przez jakieś bakterie. Czy ma ktoś jakiś pomysł? Mój mąż cierpi na raka nerki z przerzutami na węzły chłonne. Leczony na depresję,wodonercze doczekał się w/w nowotwora. W tej chwili nie ma szans na przeżycie .Waży 58 kg przy wzroście 186 cm.Je bardzo mało i też bez przerwy burczy mu w jelitach.Nie wiem o co chodzi,ale czekam na wizytę w Akademii może tam się czegoś dowiem,chociaż ostanie dni i szok jaki przeżyłam odwiedzając kolejne szpitale pozbawiły mnie już jakichkolwiek złudzeń,że w końcu ktoś zajmie się człowiekiem który przepracował sumiennie 50 lat i potrzebuje pomocy,a traktowany jest gorzej niczym zwierzę.Fakt,że należy się mam już tylko 1m ziemi nad nami to odczuwam w każdej placówce służby zdrowia. To tyle na marginesie bo mogłabym i co najmniej książkę. Pozdrawiam i życzę dużo	SubOP 14<br><br>My stomach rumbles too. I follow a diet and do not eat sugar and bloating products and it still doesn't help. I visited a gastroenterologist. He prescribed me xifaxan after that I had the trots. It helps me when I eat garlic for the night. So probably this ailment is caused by some bacteria. Does anyone have any idea? My husband has kidney cancer with lymph node metastases. Treated for depression, hydronephrosis he got above-mentioned cancer. At the moment there is no chance for survival. He weighs 58 kg with a height of 186 cm. He eats very little and it rumbles constantly in his intestines. I don't know what's going on, but I'm waiting for a visit to the [Medical] Academy, maybe I will learn something there, although the last days and the shock that I experienced visiting subsequent hospitals has deprived me of any illusions that finally someone will take care of a man who has worked diligently 50 years and needs help, and is treated worse like an animal. The fact that we should have "the only 1m of earth above us" I feel in every health care unit. That's a small thing that I wrote because I could

zdrowia i zero wizyt u lekarzy...	write at least a book. Best regards and good health and zero visits to doctors...
SubOP 15<br><br>A mi zawsze burczalo w brzuchu na spotkaniach, na lekcjach jak leze w lozku i nic. Ale 'mam srake' hahah	SubOP 15<br><br>My stomach always rumbles when I have meetings, during classes when I lay in bed and nothing. 'I have the trots' hahah
SubOP 16<br><br>ceszcie sie mi nie burczy wcale i tylko proplemy mam z zaparciami brakiem energi itd	SubOP 16<br><br>All of you should be happy. I don't have any problem with stomach rumbling but with constipation, the lack of energy, etc.
SubOP 17<br><br>Piszę ponieważ wiem jaki to problem mieć chory układ pokarmowy i że lekarze to ignoruja i zawsze tłumacza zepołem jelita wrażliwego. Rok temu miałam straszne problemy ze wzęciami, nadmiernym odawaniem gazów i biegunkami :-(było to straszne! Po wizyach u różnych lekarzy, których zaleceniem była zmiana diety i więcej sportu postanowiłam sama szukać przyczyny moich dolegliwosci, bo były tak uporczywe, że wstydziłam się spotykać ze znajomymi :-(po serii różnych badań, które zrobiłam prywatnie na własny koszt znalazłam arykuł o przeroscie flory bakteryjnej jeli i znowu ... szukanie gdzie można zrobić badania ... Całe szczęście udalo się, zrobiłam wodorowy test oddechowy i wyszedł mi znaczny przerost flory bakteryjne jelit. Po roku szukania przyczyn znalazłam ja sama i tym razem poszłam już z gotowa diagnoza do lekarza (prywatnie oczywiscie bo wizyte z nfz mialabym za 8m-cy). Dostalam antybiotyk na wyzerowanie flory bakteryjnej, kosztowal nie malo ale oplacalo sie. Po 4 miesiacach jest lepiej (moge bez stresu	SubOP 17<br><br>I write it because I know what a problem it is to have a sick digestive system and that doctors ignore it and always blame everything on the irritable bowel syndrome. A year ago I had terrible problems with bloating, excessive intestinal gases and diarrhea :-(it was terrible! After visits to various doctors whose recommendation was to change my diet and to have more sports, I decided to look for the cause of my ailments by myself, because my problems were so persistent that I was ashamed to meet with friends :-(after a series of various tests that I did privately at my own expense, I found an article about the small intestinal bacterial overgrowth and then ... I was looking at where can I do the tests ... I was lucky enough to do a hydrogen breath test and it turned out that I have a significant overgrowth of bacterial flora in the intestine. After a year of searching for the causes, I found it and this time I saw the doctor with my diagnosis (privately, of course, because for the visit financed by public health insurance, I would wait for 8 months). After 4 months I feel better (I can meet people

spotykac sie z ludzmi) chociaz nie tak jak kiedys :-(. POWODZENIA! Mam nadzieję, że może komus pomogłam:-)	without stress) although it is not like it used to be :-(. GOOD LUCK! I hope that maybe I helped someone :-)
SubOP 18<br><br>Tak jak już zostało wspomniane wyżej - często przyczyną jest coś innego niż zwykły głód, warto zadbać o równowagę flory bakteryjnej jelit. Na początek najlepiej spróbować zrównoważyć przyjmowane posiłki, jeść regularnie ale w małych ilościach - często burczy w brzuchu, bo ktoś skrupulatnie trzyma się trzech dawek dziennie o 8 rano, 13 i 18. Wtedy w przerwach nietrudno zgłodnieć, lepiej rozłożyć sobie żywienie na 5 dawek dziennych i problem znika	SubOP 18<br><br>As it was already mentioned above - often the reason is something other than just hunger, it is worth ensuring that intestinal bacterial flora is in balance. In the beginning, it's best to try to balance your meals, eat regularly, but in small quantities - your stomach often growls, because someone scrupulously eats three times a day at 8 am, 1 pm and 6 pm. Then, during breaks, it is not difficult to get hungry, it is better to eat 5 smaller portions daily and the problem disappears
SubOP 19<br><br>Len - trochę pomaga. Ja nie mam biegunek czy zaparć. Nic mnie nie boli Po prostu burczy mi w brzuchu. Spróbuję jeszcze cynamonu a jak nie to wracam do xifaxanu. Wydaj mi się, że przyczyną są bakterie albo jakieś inne świństwo / Gastrolog mówił mi że trzeba kilka razy powtórzyć kurację antybiotykową czy moga to byc pasozyty?	SubOP 19<br><br>Linseed - it helps a little. I don't have diarrhea or constipation. Nothing hurts me. I have just intestinal rumbling. I will try cinnamon and if it doesn't help, I will take xifaxan again. It seems to me that the cause is bacteria or some other bloody thing / Gastroenterologist told me that I have to repeat the antibiotic treatment several times, can I have parasites?
SubOP 20<br><br>Mam podobny problem, jak zresztą wszyscy tutaj Aczkolwiek, najbardziej uporczywe burczenie dopada mnie w trakcie leżenia, wcześniej wystarczyło, że kładłam się na boku i przestawało. A teraz nic już nie pomaga ;c .Przy tym zdarza się , że puszczam dużo bąków i dostaje skurczy brzucha(dość bolesnych). Nie mam pojęcia co mogę zrobić, żeby już nie wstydzić się spania z chłopakiem.	SubOP 20<br><br>I have a similar problem, like everyone here. However, the most stubborn rumblings I have when I lye down. So far, it was enough that I laid on my side and it stopped rumbling. Now, nothing helps anymore;c. It happens that I pump a lot and then I get stomach cramps (quite painful). I have no idea what I can do to avoid being ashamed of sleeping with my boyfriend.

SubOP 21	SubOP 21
Witam Nie będę wgłębiała się w szczegóły, ale jeszcze w styczniu wszystko było okej, nie było problemów z siedzeniem z kimś w jednym pokoju, w szkole na wykładach czy spaniu z kimś w nocy. To było jak dla innych ludzi naturalne. Natomiast po bardzo mocnym stresie, wręcz wyniszczeniu z powodu szefa, zwolniłam się, z czego miałam u niego jeszcze większe problemy. Schudłam w lutym 9 kg, ważyłam 49 kg przy wzroście 165cm. Wtedy właśnie zaczął się kompletny brak apetytu (kupiłam już apetizer), depresja, bardzo częste choroby, zimno na ustach 5 razy w miesiącu, złe samopoczucie, po małym posiłku ból brzucha, zaparcia. No i najważniejsze, to nieszczęsne burczenie. Siedziałam w biurze, a jak wiadomo tam jest cicho... burczało mi na różne sposoby, cicho, głośno, pękało powietrze, bardzo głośny pisk. Uciekałam na dół, ze stresu bolał mnie żołądek. Byłam u lekarza, podejrzewał wrzody żołądka, ale test wyszedł ujemny, więc stwierdził, że mam ZJD. Brałam przez miesiąc leki, ale gdy się skończyły, okazało się, że bez nich jest lepiej. W ogóle od stycznia unikam cichych pomieszczeń, zaczęłam teraz chodzić na studium i po prostu to jest katorga. Nie spotykam się ze znajomymi... ale spotkałam chłopaka. Jesteśmy ze sobą już 4 miesiące. Jak jesteśmy u mnie, włączam głośno tv, ale jak ma u mnie spać, albo ja u niego. Boże Święty. Mam wtedy istną burzę w brzuchu, mimo, że wie, że mam taki problem, czuję się taka spanikowana, że jak ostatnio obudziłam się w nocy, była cisza, a brzuch bez przerwy burczał, i te cholerne gazy, po prostu wyszłam w środku nocy i nie wróciłam aż do 9 rano… jak się uspokoiłam. Chłopak był mega zły, ja już nie chcę tak żyć, bo co? Będę z nim	Hello, I will not go talk about details, but in January everything was fine, I didn’t have any problems while sitting with someone in one room, at school during lectures or sleeping with someone at night. It was natural. However, after very strong stress, even emaciation because of my boss, I got off my work, and because of that, I had even bigger problems with him. I lost 9 kg in February, weighed 49 kg with a height of 165cm. Since then, I have a lack of appetite (I had already bought a drug ‘apetizer’), depression, very common diseases, herpes on my mouth 5 times a month, malaise, after a small meal, stomach ache, constipation. And the most important, I had also this horrible stomach rumbling. I was sitting in the office, and as you know it is quiet there... My stomach was rumbling in different ways, quiet, loud, air cracked, very loud squeak. I ran downstairs, my stomach hurt. I went to the doctor, he suspected stomach ulcers, but the test was negative, so he stated that I had IBS. I was taking medicine for a month, but when I ended, it turned out that it is better without them. In general, I have been avoiding quiet rooms since January, now I have started to study and it's just hard work. I don't meet my friends ... but I met a boyfriend. We've been together for 4 months. When we are at my place, I turn on the TV loudly, but what if he is going to sleep together? Holy God. I have a real storm in my stomach, even though he knows I have such a problem, I feel so panicked that the last time I woke up at night, there was silence and my belly was constantly grunting, and those damn gases, I just went out in the middle of the night and I didn't come back until 9am … when I calmed down. The boy was extremely angry, I don't want to live like that anymore,

mieszkać i uciekać co noc do innego pokoju? Nie piję napojów gazowanych, ani żadnych niegazowanych kolorowych, tylko wodę, kawę, herbatki na trawienie prawie dziennie i ewentualnie sok ale to rzadko. Nie jem w macu, żadnych fast foodów, ani smażonych rzeczy, unikam grochu i cebuli i innych takich. Staram się nie obżerać, mimo, że mam mega apetyt bo potem umieram z bólu żołądka. Macie jakieś rady? Zeby spokojnie usiąść w cichym pokoju z chłopakiem? Zeby iść na spotkanie z klientem bo teraz znów pracuję w biurze... żeby iść na wykłady bez panicznego stresu? Zeby żyć jak INNI, bez stresu, burczenia, bólu brzucha.... czasem mam wielki ból brzucha przenikający, ściskający i idę zaraz do WC się załatwić, masakra... czasem mam też jakby dniową grypę jelitową, a to nie grypa, ale może to ZJD. Nie wiem już co robić, jestem jednym wielkim kłębkiem nerwów.	because.. Will I live with him and run to another room every night? I don't drink carbonated or non-carbonated colored drinks, only water, coffee, teas for digestion almost every day and possibly juice, but this is rare. I don't eat in McDonald's, I eat no fast food or fried things, I avoid peas and onions and any different food like that. I try not to overeat, even though I have a bit of appetite because then I am dead because of a stomach ache. Do you have any advice? Advice, that could allow me to sit back in a quiet room with my boyfriend? To go to a meeting with a client because now I work in the office again … or to go to lectures without panic and stress? To live like OTHERS, without stress, rumbling, stomach ache .... sometimes I have a great stomach ache to penetrate, squeezing and I go to the toilet to plop, horrible ... sometimes I have a kind of intestinal flu, which is not flu but maybe it's irritable bowel syndrome.. I don't know what to do anymore, I'm a big bundle of nerves.
SubOP 22<br><br>ja nie mam żadnego rozwolnienia, ani żadnych bólów brzucha, za to właśnie to przelewanie się i burczenie doskwiera mi o każdej porze dnia. I to nie jest z głodu, ponieważ najczęściej pojawia się chwilę po zjedzeniu albo wypiciu czegoś. Ten problem mam już od roku, zauważyłam, że pojawił się przy zmianie diety, ale gdy wróciłam do starego zwyczaju żywieniowego problem nie zniknął. Jedna istotna rzecz, która zauważyłam to, że najczęściej burczy mi po wypiciu kawy, tak więc to też może być z tego powodu, jak odstawiłam picie kawy z rana to już nie miałam, aż tak głośnego burczenia, ale problem całkowicie nie zniknął. Nie mam zielonego pojęcia co z tym zrobić a do lekarza za bardzo też nie mogę się wybrać,	SubOP 22<br><br>I don't have any diarrhea or stomach ache, but this rumbling bothers me at any time of the day. And this is not hunger, because it usually appears a moment after eating or drinking something. I had this problem for a year, I noticed that it appeared when I changed my diet, but when I returned to the old eating habits the problem did not disappear. One important thing that I noticed was that I have this rumbling usually after drinking coffee, so it could also be a cause. When I stopped drinking coffee in the morning, I didn't have that loud rumble, but the problem did not disappear completely. I have no idea what to do with it. What’s more, I can't see the doctor because I moved to Germany lately and I don't know the language so good that I

ponieważ nie dawno przeprowadziłam się do Niemiec i nie znam na tyle języka, aby porozmawiać o tym z jakimś specjalistą.	could talk to a specialist about my problem.
SubOP 14<br><br>Mój mąż cierpi na raka nerki z przerzutami na węzły chłonne. Leczony na depresję,wodonercze doczekał się w/w nowotwora. W tej chwili nie ma szans na przeżycie .Waży 58 kg przy wzroście 186 cm.Je bardzo mało i też bez przerwy burczy mu w jelitach.Nie wiem o co chodzi,ale czekam na wizytę w Akademii może tam się czegoś dowiem,chociaż ostanie dni i szok jaki przeżyłam odwiedzając kolejne szpitale pozbawiły mnie już jakichkolwiek złudzeń,że w końcu ktoś zajmie się człowiekiem który przepracował sumiennie 50 lat i potrzebuje pomocy,a traktowany jest gorzej niczym zwierzę.Fakt,że należy się mam już tylko 1m ziemi nad nami to odczuwam w każdej placówce służby zdrowia. To tyle na marginesie bo mogłabym i co najmniej książkę. Pozdrawiam i życzę dużo zdrowia i zero wizyt u lekarzy...<br><br>Konowały w każdym szpitalu wiedzą że jak się ruszy nowotwór to w ciagu góra 2 miesięcy chory umiera. Ale mimo to rzucają na stół operacyjny i kroją bo nfz nie będzie płacił za leczenie bezoperacyjne zw£aszcza jeżeli chodzi o starszych ludzi na emeryturach.Właśnie zmarła mi mama ,poszła do szpitala bez żadnych bóli, czuła się dobrze,zrobiła jej się przetoka. Z przetoką żyła bez innych dolegliwości pół roku, zgłosiła się tylko zewzględu na dyskomfort,szybkie badania,wykrycie guza na jelicie i po dwóch dniach operacja,a cztery dni po operacji kolostomi "kopa" do domu. Już z łóżka nie wstała,życie w niej gasło z godziny na godzine, po czterech tygodniach zrobiła się zakrzepica i martwica stopy,dosz£a	SubOP 14<br><br>My husband has kidney cancer with lymph node metastases. Treated with depression, hydronephrosis he got above-mentioned cancer. At the moment there is no chance for survival. He weighs 58 kg with a height of 186 cm. He eats very little and it rumbles constantly in his intestines. I don't know what's going on, but I'm waiting for a visit to the Academy, maybe I will learn something there, although the last days and the shock that I experienced visiting subsequent hospitals has deprived me of any illusions that finally someone will take care of a man who has worked diligently 50 years and needs help, and is treated worse like an animal. The fact that we should have “only 1 m of earth above us” I feel in every health care unit. That's a small thing that I wrote because I could write at least a book. Best regards and good health and zero visits to doctors ...<br><br>Those quacks in every hospital know that if the cancer is in progress, within 2 months the patient dies. But, despite this, they throw the people on the operating table and cut because the National Health Fund will not pay for non-surgical treatment, especially when it comes to elderly people in retirement. My mother just died, she went to the hospital without any pain, she felt good, she had a fistula. With a fistula, she lived without any other ailments for half a year, she visited a doctor only because of discomfort. Then, she had a rapid examination, and the tumor of the intestine was detected. After two days she had surgery, and four days after the surgery (colostomy), she was "kicked" home. She

amputacja nogi. Zmarła po 7 tygodniach. Pies jeb...ał naszą służbę zdrowia.	didn't get off a bed anymore, her life was extinguishing hour by hour. After four weeks she had thrombosis and foot necrosis, then amputation of her leg. She died after 7 weeks. Fu*** ... our health service.
SubOP 23<br><br>Mam podobny problem wstaję do szkoły na 7 i jem śniadanie jakąś kanapkę itp. Zawsze jak wstaję od stołu zaczyna się bulgotanie i przelewanie jak spałam u koleżanki to miałam jeszcze gorzej byłyśmy same w domu i burczało mi tak jakby ktoś przelewał wodę okazało się że ona ma podobny problem na internecie znalazłyśmy masarż jelit i ona zrobiła go mi a ja jej i pomogło ale później z łazienki nie wychodziłam 4 godziny pomocy !!!	SubOP 23<br><br>I have a similar problem, I get up to school at 7 and eat breakfast, a sandwich, etc. Whenever I get up from the table, it starts bubbling and splashing. When I slept at my friend's, it was worse, we were alone at home and I had this rumbling. It sounded as if someone was pouring water. It turned out that she had a similar problem, on the internet, we found a bowel massage and she did it to me and I did it to her. It helped us but later I couldn't leave the bathroom for 4 hours, help !!!
SubOP 24<br><br>Masz uczucie pełności i uczucie wzdęcia?	SubOP 24<br><br>Do you have a feeling of being full or some flatulence?
SubOP 25<br><br>Czy moga to byc pasozyty? przyczyna burczenia w brzuchu moga byc pasozyty!! Ktory lekarz i gdzie moze nas z tego wyleczyc?	SubOP 23<br><br>Is it possible that I have parasites? Parasites could be a cause of stomach rumbling!! Which doctor and where can heal us?
SubOP 25<br><br>witam ,mam podobny problem ,wdaje mi się ze to wszystko ma powiazanie z przerostem grzyba zwanego "candida" pasożytniczego w jelitach, który pozera wszystkie cenne składniki w celu zaspokojenia swych potrzeb gdy mu brakuje drazni jelito w celu dostarczenia nowych skladnikow,zauwazcie ze zaraz po zjedzeniu wszystko wraca do normy .pierwszym sposobem zauważenia rezultatow wezcie się za sprowadzenie tego grzyba do przyzwoitej ilości ,inaczej nic nie pomoze .niech każdy dowie się jaka diete zastosować by go obezwladnic i	SubOP 25<br><br>hello, I have a similar problem, it seems to me that all of this is related to the overgrowth of the parasitic fungus called "candida" in the intestines, which eats up all of the valuable ingredients to meet its needs. When it lacks some ingredients it irritates intestine to provide new ones. You could notice that immediately after eating, everything returns to normal. the first way to notice the results is to reduce the amount of these fungi to a decent amount, otherwise, nothing helps. let everyone find out what diet to use to overpower it and

ograniczyć mu rozrost w początkowych fazach ,później sprowadzić jego populacje do rozmiaru jaki powinien być w jelitach ,inaczej nie osiagniecie nic. Nie wiem dlaczego lekaze maja po prostu wszysko gleboko w du*** ,liczy się dla nich tylko kasa jak pojdziesz nawet prywatnie ,szacunek zerowy ,	how to limit its growth in the initial stages, then reduce its populations to the proper size that should be in the intestines, otherwise, you will not achieve anything. I do not know why doctors give a sh** about it, they care only about money, even when you go for the private visit they don't have any respect
SubOP 26<br><br>Ludzie napiszę wam tak: pierwsza sprawa to odrzucić kawę, jest to kwas niszczący śluzówki jelitowe, druga sprawa, musicie pić dwa, trzy razy dziennie szałwie, po tygodniu dopiero zauważcie poprawę waszych jelit, po dwóch miesiącach przestanie wam burczec w odbycie, po trzech miesiącach w jelitach, teraz tylko szałwia na czczo raz dziennie rano. Trzecia sprawa musicie jeść chleb pszenny bez żadnych dodatków, raz dziennie jogurt naturalny. Chleb smarować tylko i wyłącznie majonezem. Żadnych smalcow, margaryny, masła. Czwarta sprawa, żadnych słodyczy i ciastek, zero piwa i gazowanych napoi. Po czterech miesiącach rezygnujecie z szałwii i przechodzicie na rumianek, pijecie raz dziennie przez dwa miesiące, zaraz po wypiciu musicie coś zjeść. Rezygnujecie z jogurtu naturalnego gdy pijecie rumianek, rumianek by złagodzić objaw głodu pić z łyżeczką miodu. Po sześciu miesiącach macie wyleczone jelita. Teraz zostało wam tylko burczenie w żołądku i będzie tylko wtedy gdy będziecie głodni. Po pół roku od stosowania tych czynności odrzucacie rumianek. Teraz pomału wracacie do zdrowia, jeżeli jednak zaczniecie na nowo pchać w siebie czekoladę, czipsy i pompowac się kawą to niestety klapa nastąpi jak się domyślacie. Aha żadnych leków gdy to będziecie stosować, oprócz najwyżej jak zajdzie potrzeba raz na jakiś	SubOP 26<br><br>People, I will write it to you like this: the first thing is to reject coffee, it is acid that destroys intestinal mucosa, the second thing is, you have to drink salvia two or three times a day, after a week you will notice the improvement of your intestines, after two months it will stop rumbling in a rectum, after three months in the intestines, now, you drink only salvia once a day in the morning when you are fasting. The third thing you must eat wheat bread without any additives, natural yogurt once a day. Spread bread only with mayonnaise. No lard, margarine, butter. Fourth thing, no sweets and cakes, no beer and carbonated drinks. After four months, you give up salvia and you go for chamomile, drink once a day for two months, immediately after drinking you have to eat something. You give up natural yogurt when you drink chamomile. To relieve hunger while drinking chamomile, have a teaspoon of honey with it. After six months you will have your intestines healed. Now, all you have is a stomach grunting you will have this problem only when you are hungry. After six months of using these things, you reject camomile. Now you are slowly recovering, but if you start to eat chocolate, chips and pump coffee again, unfortunately, all that you have done would be nothing, as you guess. Oh, and you can't take any drugs when you use this therapy, except for some painkillers that you need from time to time. Prophylactically,

czas można wziąć proszki przeciwbólowe. Profilaktycznie przeciw chorobom raz, dwa razy w tygodniu zjeść czosnek, cebulę, mogą pojawić się objawy wzdęcia i burczenia ale nie wpłynie to na ogólną kurację jelit. Pozdrawiam.	against diseases, you can eat garlic and onions once or twice a week, there may be some symptoms of bloating and rumbling but this will not affect the overall bowel treatment. Best regards.

OP – original poster, subOP – suboriginal poster

In this thread, the OP complained about rumbling in her stomach. She was afraid of seeing a doctor because she did not want to have a gastroscopy.

The forum users recommended performing gastroscopy and even colonoscopy, ensuring that there is no need for fear. The OP received also some information about eating habits that can soothe the ailments. The responders advised also to stick to some unconventional methods such as eating garlic, linseed, salvia, cinnamon or calamus brew as well as using some specific drugs (e.g. Xifaxan, Espumisan, Duspatalin Retard) or tests toward parasites, fungi or small intestinal bacterial overgrowth.

Many responders wrote also about their problems, looking for help and emotional support, some of them just react mockingly to problems of others. It is worth to mention that some users slandered doctors for their incompetence or bad treatment. There were also proposals for long-term unconventional treatment that were described by responders.

The presented example reflects that outside the medical community, there are plenty of unconventional methods for treatment. It can complement medical therapy. However, it can also pose a risk that interferes with conventional medicine, especially when some forum users discourage others from seeing a doctor and encourage to self-treatment with prescription drugs.

E. Record ID 1315

Polish (original spelling)	English translation
Tytuł: Krew w kale u 4 letniego dziecka i brak innych objawów	Title: Blood in stool in 4-year-old child and the lack of other symptoms
Initial post (OP)<br><br>Witam. Bardzo proszę o wskazówki co może byc przyczyną krwi widocznej w kale u mojego 4 letniego synka, często robi normalne stolce z krwia i śluzem, czasem boli go lekko brzuszek z lewej strony pod żebrami (w czasie wypróżniania). Alergia i hemoroidy raczej wykluczone. Pasożyty też wyklucone. Sytuacja utrzymuje się już 4 miesiące, a dotychczasowe badania nic konkretnego nie wykazały. morfologia w normie oprócz pozimou żelaza (wynik 25) i mikrocytoza erytrocytów, mocz w normie, OB podwyższone (12), fibrynogen podwyższony (710), ALT, AST GGTP, glukoza, albumina czas protrombinowy, czas kaolinowo-kefalinowy w normie, posiew stolca ujemny, pasożyty ujemne, krew utajona dodatnia, usg prawidłowe, gastroskopia prawidłowa, kolonoskopii nie zrobili, bo jest kolejka. Z takimi wynikami odesłali nas ze szpitala i nic konkretnego nie powiedzieli. poza tym synek jest normalny, zdrowy, nic go nie boli, pełny energii. miewa czasem bóle stawów - kolana i staw skokowy, ale jakis czas temu ortopeda orzekł, że to bóle wzrostowe, żadnych badań nie wykonywał. w szpitalu przy przyjęciu stwierdzili też powiększone węzły chłonne na szyi mimo, że nie był przeziębiony, ale chodzi do przedszkola, więc może coś tam bezobjawowo podłapał. Proszę o wskazówki na co takie wyniki wskazują, jakie powinnien mieć zrobione badania. Z góry dziękuję.	Initial post (OP)<br><br>Hello. I am asking for tips on what may be the cause of blood visible in the feces of my 4-year-old son, he often makes normal stools with blood and mucus, sometimes he has a slight stomachache under the ribs (during defecation). Allergy and hemorrhoids are rather excluded. Parasites too. The situation has persisted for 4 months, and previous studies have not shown anything significant. Peripheral morphology is within normal range, except of iron levels (value of 25) and erythrocytes microcytosis, normal urine, elevated ESR (12), elevated fibrinogen (710), ALT, AST GGTP, glucose, albumin, prothrombin time, normal kaolin-cephalin clotting time, stool culture-negative, negative parasites, positive occult blood, normal ultrasound, normal gastroscopy, colonoscopy wasn’t done, because there is a long queue. With such results, we were discharged from the hospital and without a specific diagnosis. in addition, the staff said that the son is normal, healthy, nothing hurts him, he is full of energy. sometimes he has joints pain - knees and ankle, but some time ago an orthopedist stated that it was growth pain, he did not do any tests. in the hospital, they also found enlarged lymph nodes in the neck, even though he wasn't having a cold, but he was going to kindergarten, so maybe he becomes infected with something asymptomatically. I am asking for tips on what such results indicate and what tests my son should do. Thanks in advance.

SubOP1<br><br>Musicie zrobić kolonoskopie. Jedyne badanie które konkretnie stwierdzi co jest grane. Moze jakies wrzody jelita grubego lub cos innego. Rowniez mozna spróbować z tomografia.	SubOP1<br><br>You have to perform a colonoscopy. This is the only test that will tell you what's wrong. Maybe some colon ulcers or something else. You could also try to perform computed tomography.
SubOP2<br><br>No to fajnie zdiagnozowali dzieciaka. Krew leci ale generalnie to do domu. U moich pacjentów przy bolących kolanach i niżej , testuje się chlamydia trachomatis. To typowe objawy. Tradycyjne metody usuwania tego świństwa są mało skuteczne i toksyczne. Zaleczanie dotyczy głównie ukł płciowego. Bóle wzrostowe dotyczyć powinny równiez reszty ukł kostnego np kręgosłupa A dlaczego niby dziecko nie miałoby mieć pasożytów? Bo napisali , że test nie wykazał ? A napisali że skuteczność badania kału na tasiemca to tylko 10-15 % ? Naprawdę do testów należy podchodzić z dystansem. Mogą świadczyć o wszystkim i o niczym. Węzły chłonne powiększone ? A od jakiego czasu ? A może jakieś podstawowe testy powinni zrobić ? Oj NFZ nie ma kasy już wyraźnie. Pozdrawia Terapeuta ps W razie jakiś pytań kontakt przez nick bo nie wracam do postów	SubOP2<br><br>Well, nice diagnosis. There is blood in stool but generally, they discharged him out of the hospital. Among my patients with aching knees and below, chlamydia trachomatis is tested. These are typical symptoms. Traditional methods of removing Chlamydia are not very effective and can be toxic. Healing mainly concerns the reproductive system. Growth pains should also concern the rest of bones, e.g. the spine. Why shouldn't a child have parasites? Because they wrote that the test did not show? Did they write that the effectiveness of testing feces to diagnose taeniasis is only 10-15%? You really need to be careful when it comes to those tests. Those results are not meaningful. Lymph nodes enlarged? How long? Or maybe they should do some basic tests? Oh, National Health Fund has no money, obviously. Best regards from The Therapist. ps If you have any questions contact me by nick because I do not return to posts
SubOP 3<br>Gabinet biorezonansu bio-klar.pl (likwidacja alergii, pasożytów, bakterii, ...	SubOP 2<br>bio-klar.pl bioresonance office (elimination of allergies, parasites, bacteria, ...
OP2 (OP update)<br><br>Udało się zrobić kolonoskopię. Okazało się, że jest polip z owrzodzeniem o śr. ok.2,5cm. W naszym szpitalu nie mają sprzętu do usunięcia takiego dużego polipa więc musimy jechać do CZD, mamy bardzo szybki termin, bo czekamy tylko	OP2 (OP update)<br><br>The child had a colonoscopy. It turned out that there is a polyp with ulceration about 2.5 cm in diameter. In our hospital they do not have the proper equipment to remove such a large polyp, so we have to go to Child Health Center, we have a very short

tydzień od zgłoszenia.	appointment because we wait only a week from reporting the problem.

OP – original poster, subOP – suboriginal poster

This thread concerns the problem with the blood and mucus in the stool of a 4-year old child and its stomach ache. The OP wrote that some laboratory tests were performed as well as stool culture, parasite testing, ultrasound, and gastroscopy. Some of the results were beyond the normal ranges: lower iron level in serum, microcytosis, elevated ESR and fibrinogen. The OP also mentioned joint pain in her child but was told by doctors, that it was just a growth pain.

One of the responders immediately recommended performing the colonoscopy. However, there was also a response that suggested the parasite testing could give false-negative results because of its low sensitivity or that the joint pain could be caused by C. Trachomatis. In this way, subOP2 slandered the treatment proposed by doctors. Additionally, the same person who gave above-mentioned tip advertised his own services such as „bioresonance office".

Finally, a colonoscopy revealed that the child had a single polyp in the colon. This case shows that advices on an Internet forum can be misleading and potentially harmful.

Some users may try to manipulate other people, to show that their services are more effective than conventional medicine. They often use convincing arguments to achieve their goals. Fortunately, the OP chose a visit to a doctor.

F. Record ID 2460

Polish (original spelling)	English translation
Tytuł: Bardzo szybka utrata masy ciała a wrzody na żołądku	Title: Very fast weight loss and stomach ulcers
Initial post (OP)<br><br>Witam. Mam 21l i rok temu przechodziłem chorobe wrzodowa. Wszystko było okej do dziś. Od 16.03.16 utraciłem 4kg nogi rece wszystko strasznie schudło nie mam apetytu i ciagle burczy mi w brzuchu. Prosze o szybko odpowiedz.	Initial post (OP)<br><br>Hello. I am 21 years old and I had been suffering from peptic ulcer disease a year ago. Everything was ok until today. Since 16/03/16 I have lost 4 kilograms. My legs, my arms, everything lost a lot of weight, I have no appetite and my stomach is still rumbling. Please answer quickly.
SubOP1<br><br>To do lekarza a nie czekać na forum na poradę!	SubOP1<br><br>See a doctor instead of waiting on the forum for the advice!
SubOP 2<br><br>Na dzień dobry posłuchaj rady medynara. A dodatkowo to masz faktycznie chory układ pokarmowy. Nie szanowałeś go , czy jakiś pasożyt ? Niechybnie uszkodzony odźwiernik żołądka. Niska kwasowość żołądka. Zła dieta rozwaliła Ci opuszkę i w żołądku zapewne rozwinęła Ci się Helicobacter Skoro chudniesz to kuleje Twoja przyswajalność i do obejrzenia jest dwunastnica i jelita. Do zbadania celiaklia albo testy na pasożyty dwunastnicy i okolicy Coś czuję , że mi się tu rymuje Dużo pisania. W razie pytań kontakt przez nick , bo nie wracam do postów przeczytanych Pozdrawia Naturopata	SubOP 2<br><br>In the beginning, you should listen to medinar's advice. Also, you actually have a sick digestive system. Did you not respect it or you have some parasite? Inevitably, you have damaged stomach pylorus and low stomach acidity. A bad diet has smashed a 'bulb' and Helicobacter probably developed in your stomach. If you lose weight, your digestibility is worse and worse and the duodenum and intestines should be tested. You should perform celiac disease tests or tests for parasites in the duodenum and the surrounding area. I feel that I rhyme when I write a lot. If you have questions, contact by nick because I do not go back to posts. Greetings from Naturopata
SubOP 2<br><br>Gabinet biorezonansu bio-klar.pl (likwidacja alergii, pasożytów, bakterii, ...)	SubOP 2<br><br>Bio-klar.pl bioresonance office (elimination of allergies, parasites, bacteria, ...)

OP – original poster, subOP – suboriginal poster

The OP with the history of stomach ulcers, reported a problem with sudden weight loss, lack of appetite and stomach rumbling.

The first answer suggested seeing a doctor, which was obviously right. However, the second response suggested that the OP has a parasite and bacterial infection or insufficiency of a digestive system. The post itself is very encouraging and invites contact. The third response was an advertisement for bioresonance office. Nonetheless, there is no evidence to follow the advice of the user „Naturopata" or to use bioresonance. Such offers should be filtered and deleted on a medical forum.

Table S4

Univariate regression analysis. Dependent variable: the initial post with at least one harmful advice (coded as one) vs. initial post with non-harmful advice(s) (coded as null).

Independent variable	OR (95% CI)	p-value
Question about treatment of declared ailment	0.75 (0.48-1.19)	0.22
Question about diagnostics of declared ailment	1.27 (0.77-2.1)	0.33
Request for diagnostics results interpretation	1.53 (0.74-3.17)	0.27
Request for drug/doctor/healthcare facility recommendation [diagnosed disease]	1.64 (0.56-4.75)	0.39
Request for emotional support and/or motivation	0.39 (0.09-1.61)	0.13
Complexity of the problem	1.27 (0.82-1.98)	0.28
Duration of the problem (> four weeks)	1.13 (0.96-1.34)	0.14
Abdominal pain	1.15 (0.74-1.79)	0.54
Ascites	0 (0-Inf)	0.34
Anus itch	1.25 (0.29-5.45)	0.77
Anus lesion	0 (0-Inf)	0.14
Back pain	1.73 (0.51-5.88)	0.41
Belching	1.16 (0.45-2.99)	0.76
Bloating	1.56 (0.91-2.7)	0.12
**Blood in stool**	**6.21 (3.75-10.3)**	**< 0.001**
Chest pain	2.24 (0.64-7.81)	0.25
**Constipation**	**1.80 (1.04-3.12)**	**0.046**
Diarrhea	1.50 (0.91-2.47)	0.12
**Dyspepsia**	**3.69 (1.63-8.33)**	**0.005**
**Dysphagia**	**7.71 (1.81-32.84)**	**0.015**
Fatigue	1.47 (0.71-3.04)	0.32
Feeling of incomplete defecation	1.64 (0.56-4.75)	0.39
Fever	0.83 (0.11-6.35)	0.85
Flatulence	0.90 (0.44-1.84)	0.70
Halitosis	0 (0-Inf)	0.03
**Headache**	**2.60 (1.05-6.44)**	**0.06**
Heartburn	1.61 (0.71-3.64)	0.28
Hemoptysis	3.14 (0.35-28.43)	0.37
**Hypogastrium region pain**	**2.34 (1.11-4.93)**	**0.04**
Intestinal rumbling	1.20 (0.58-2.47)	0.63
Jaundice	0 (0-Inf)	0.50
**Left lower quadrant pain**	**3.19 (1.35-7.5)**	**0.017**
Light upper quadrant pain	0.94 (0.37-2.4)	0.90
Loss of an appetite	0.56 (0.13-2.33)	0.38
Nausea	0.94 (0.46-1.92)	0.86
Odynophagia	0 (0-Inf)	0.50

**Rectal or anal pain**	**2.19 (1-4.79)**	**0.07**
**Right lower quadrant pain**	**4.72 (2.22-10.04)**	**< 0.001**
Right upper quadrant pain	0.96 (0.4-2.26)	0.92
Subfebrile	1.04 (0.13-8.09)	0.97
Umbilical region pain	1.53 (0.64-3.68)	0.36
Vomit	1.01 (0.39-2.57)	0.99
**Weight loss**	**5.44 (2.68-11.02)**	**< 0.001**
Weight gain	0 (0-Inf)	0.30
Anal fissure	4.19 (0.43-40.75)	0.28
Anemia	27037944.41 (0-Inf)	0.001
Anxiety disorder	0 (0-Inf)	0.34
Arterial hypertension	2.09 (0.25-17.57)	0.53
Bile reflux	0 (0-Inf)	0.69
Biliary tract diseases	6.30 (0.57-70.14)	0.19
Celiakia	0 (0-Inf)	0.38
Chronic pancreatitis	0 (0-Inf)	0.69
Depression	6.30 (0.57-70.14)	0.19
Diabetes	0 (0-Inf)	0.50
Duodenitis	0 (0-Inf)	0.43
Esophageal varices	0 (0-Inf)	0.69
Esophagitis	0 (0-Inf)	0.50
Gastroenteritis	0.73 (0.17-3.08)	0.65
Gastrointestinal allergies and intolerances	0 (0-Inf)	0.38
Gastrointestinal fungal disease	0 (0-Inf)	0.43
Gastroesophageal reflux disease	0 (0-Inf)	0.69
Helicobacter pylori infection	0.73 (0.1-5.55)	0.75
Hemorroids	2.00 (0.58-6.91)	0.31
Hiatal hernia	0 (0-Inf)	0.094
**Hypothyroidism**	**9.65 (2.13-43.84)**	**0.009**
**Inflammatory bowel disease**	**5.50 (1.4-21.66)**	**0.033**
Irritable bowel syndrome	1.54 (0.53-4.44)	0.45
Liver cirrhoris	0 (0-Inf)	0.50
Malignancy	9831031.92 (0-Inf)	0.022
Overweight or obese	1.79 (0.22-14.72)	0.61
Parasites	4.19 (0.43-40.75)	0.28
Pregnancy	2.08 (0.25-17.5)	0.54
Peptic ulcer disease	0 (0-Inf)	0.27
Viral hepatisis	12.6 (0.78-203.27)	0.11

CI – Confidence Interval; Inf – Inifinite; OR – Odds Ratio

Table S5

Duplicated posts.

No.	Polish (original spelling)	English translation
1.	"Jak tu nie uzyskasz odpowiedzi to jest wiele portali medycznych gdzie można uzyskać odpowiedź,wystarczy wpisać w google, forum medyczne, i gdzieś na pewno uzyskasz odpowiedź"	„If you here do not find an answer there are multiple medical portals where you may find an answer, just type in google, forum medyczne, and somewhere you must obtain the answer"
2.	"Może to braki w florze bakteryjnej. Ja w takich przypadkach biorę trilac bo to sprawdzony probiotyk"	„You may have a deficiency of bacterial microflora. If I were you, I would like to take trilac because it is valid probiotic"
3.	"Zapytaj może na portalu medyczne-forum gdzie są specjaliści.Jak już korzystałam dlatego polecam.Pozdrawiam"	„Maybe ask on portal medyczne-forum where you may find specialists. I have already used this forum that's why I recommend it. Greetings"
4.	"Może spróbuj z jakimiś dobrym probiotykiem np. trilac"	„May try out a good probiotic for instance trilac"
5.	"Polecam portal medyczne-forum gdzie są specjaliści.Jak już korzystałam dlatego polecam.Pozdrawiam"	„I recommend portal medyczne-forum where you may specialists. I have already used this forum that's why I recommend it. Greetings"
6.	"Na biegunki moim zdaniem dobry jest trilac IBS. Ostatnio wzięłam podczas biegunki i bardzo pomógł. Znacznie lepszy niż tragiczny stoperan który tylko zatrzymuję stolec w jelitach"	„In my opinion on diarrhea good is trilac IBS. Recently, I took [trilac] during diarrhea and it helps me. [Trilac] is significantly better than tragic stoperan which only stop stool in the intestine"
7.	"Szukaj forum gdzie są lekarze, tu nie ma żadnego lekarza. W wyszukiwarce znajdziesz wiele portali medycznych gdzie można uzyskać odpowiedź,wystarczy wpisać w wyszukiwarkę, forum medyczne, i gdzieś na pewno uzyskasz odpowiedź"	„Search for a forum where you may find doctors, there is no doctor. In search engine you may find multiple medical portals where you may find an answer, just type in a search engine, medical forum and somewhere you must obtain an answer"
8.	"Jeśli oczkujesz profesjonalnej odpowiedzi, szukaj forum gdzie są	„If you expect professional answer search for a forum with doctors, there

	lekarze, tu nie ma żadnego lekarza. W wyszukiwarce znajdziesz wiele portali medycznych gdzie można uzyskać odpowiedź,wystarczy wpisać w wyszukiwarkę, forum medyczne, i gdzieś na pewno uzyskasz odpowiedź"	is no doctor. In search engine you may find multiple medical portals where you may find an answer, just type in a search engine, medical forum and somewhere you must obtain an answer"

9.	"Jeśli oczkujesz profesjonalnej odpowiedzi, szukaj forum gdzie są lekarze, tu nie ma żadnego lekarza. W wyszukiwarce google znajdziesz wiele portali medycznych gdzie można uzyskać odpowiedź,wystarczy wpisać w wyszukiwarkę, forum medyczne, i gdzieś na pewno uzyskasz odpowiedź"	„If you expect professional answer search for a forum with doctors, there is no doctor. In google search engine you may find multiple medical portals where you may find an answer, just type in a search engine, medical forum and somewhere you must obtain an answer”

10.	"Jak tu nie uzyskasz odpowiedzi to jest wiele portali medycznych gdzie można uzyskać odpowiedź,wystarczy wpisać w wyszukiwarkę, forum medyczne, i gdzieś na pewno uzyskasz odpowiedź"	„If you do not find an answer there are multiple medical portals where you may find an answer, just type in search engine forum medyczne, and somewhere you surely will obtain an answer"
11.	"Witam Jeśli chodzi o interpretacje wyników to jest problem, bo w polskim internecie nie ma narzędzia do sprawdzania wyników badań - do czasu Z tego co wiem na znanywynik.pl można wpisać swój wynik i do tego jest interpretacja lekarska i książkowa. To nie jest spam, bo mam pewność że, tam na pewno uzyskasz pomoc. Pozdrawiam."	„Hello If it comes to the interpretation of the results it's a problem because in the Polish Internet there are no tools to analyse the results of the diagnostics – to date. I know that in a website znanywynik.pl you may type your outcome and you obtain doctor's interpretation and interpretation from a book. This is not spam because I am sure that you there find help. Greetings"
12.	"Brzydki zapach z ust to lamblioza. Proszę sprawdzić testem . Ale enzymatycznym (płatny) bo zwykły polegający na oglądaniu kału jest nieskuteczny Pozdrawiam i współczuję Terapeuta"	„Bad smell from the mouth is lambliosis. Please check it by a test. But enzymatic (payable) because of an ordinary microscopic test is insufficient. Greetings and I am sorry Terapeuta"
13.	"Może to być zaparcie, ja stosuje na problemy trawienne i jelitowe trilac można zapytać w aptece mi zawsze pomaga."	„It could be constipation, I use for gastric problems trilac you ma ask in pharmacy it always help me"
14.	"Zespół jelita nadwrażliwego w skrócie IBS to choroba przewlekła przewodu pokarmowego charakteryzuje się bólami brzucha i zaburzeniami rytmu wypróżnień, jakiś czas temu zdiagnozowali to u mnie, to poważny problem dotyczy głównie kobiet problemy żołądkowe gazy biegunka wzdęcia, ostatnio polecono mi trilac IBS preparat pomagający w leczeniu tej dolegliwości , kupiłam i stosuję od jakiegoś czasu zauważam poprawę mam nadzieję że wszystko będzie ok."	„Irritable bowel syndrome in brief IBS it is a chronic disease of gastrointestinal tract characterized by abdominal pain and impairment of defecation rhythm, some time ago it was diagnosed with me, it is a serious problem and mainly concerns women gastric ailments flatulence diarrhea bloating, recently I was recommended trilac IBS specimen that helps in treatment of this disorder, I bought it and I use it for some time now I noticed an improvement I hope that everything will be OK."

15.	"ja stosuje na problemy trawienne i jelitowe trilac można zapytać w aptece mi zawsze pomaga."	„I use for gastric problems trilac you may ask in pharmacy it always helps me”
16.	"Sprawdzić i może zastosować jakąś dobrą dietę, może to tez być zespół jelita nadwrażliwego proponuję poczytać o tym spytać lekarza. Moja ciocia która jest samotna i jej pomagam ma podobny problem stosuje środek trilac IBS który można włączyć do diety jest poprawa."	„Check and follow a good diet, it may be also irritable bowel syndrome I recommend you read about it and ask your doctor. My aunt which is a lonely person and I help her has a similar problem and she uses trilac IBS that may be included to diet it improves”
